# *In Vivo* Murine Model of Leukemia Cell-Induced Spinal Bone Destruction

**DOI:** 10.1155/2017/3521481

**Published:** 2017-09-28

**Authors:** Jia-Jie Chen, Wei Zhou, Nan Cai, Gang Chang

**Affiliations:** ^1^Institute of Molecular Medicine, Health Science Center, Shenzhen University, Shenzhen 518060, China; ^2^Vaccine Research Institute of Sun Yat-sen University, The Third Affiliated Hospital of Sun Yat-sen University, Guangzhou, China; ^3^Department of Spine Surgery and Joint Surgery, The Third Affiliated Hospital of Guangzhou Medical University, Guangzhou 510150, China

## Abstract

Osteolytic bone lesions can be a consequence of leukemic bone infiltration or focal bone destruction by inflammatory factors released from leukemic cells. Destructive bone lesions have a negative impact on the quality of life of leukemia patients, causing unbearable pain and, in some cases, limb paralysis. However, the mechanism, by which leukemic cells produce destructive bone lesions, and the effect of therapeutics on osteolytic lesions have not been fully elucidated yet and, thus, stand to benefit from an* in vivo* model. To that end, HL-60 cells were transformed by retrovirus-mediated constitutively active (CA) STAT5 expression and injected into nonobese diabetic (NOD)/SCID mice via the tail vein. After three weeks, lumbar spines were subjected to histocytometric analysis. Xenograft mice developed hind limb paralysis in 2-3 weeks, which was consistent with the consequences of spinal bone destruction by extramedullary invasion of leukemia cells. The* in vivo* model will improve the understanding and treatment of osteolytic bone lesions caused by myeloid leukemic cells.

## 1. Introduction

Myeloid leukemia (ML) is a clonal myeloproliferative hematopoietic stem cell disorder [1]. Aside from those patients with chronic myeloid leukemia (CML) induced by wild-type BCR-ABL fusion protein or those with acute promyelocytic leukemia (APL) caused by reciprocal chromosomal translocation of the retinoic acid receptor *α* (RAR*α*) gene, most patients have a poor prognosis [2, 3]. In the United States, the 5-year survival rate of patients with acute myeloblastic leukemia (AML) is only approximately 26% [4]. Aside from more common complications such as bleeding and infection, AML patients may suffer from bone marrow necrosis (BMN). BMN is a histopathologic diagnosis characterized by destruction of the medullary stroma with preservation of cortical bone. Severe BMN occurs in about 2.4% of AML [5]. The overall survival of AML with BMN is 3.7 months compared to 14 months in those without BMN [6].

Osteolytic bone lesions can be a consequence of leukemic bone infiltration or focal bone destruction by inflammatory factors released from leukemic cells [7]. With the exception of multiple myeloma, lytic lesions are rarely associated with hematologic malignancies. Moreover, destructive bone lesions have a negative impact on the quality of life of leukemia patients, causing unbearable pain and, in some cases, limb paralysis [8]. Osteoarticular changes may occur in up to 23% of cases of acute lymphoblastic leukemia (ALL) and even more frequently in AML [9]. Previously reported clinical cases of destructive osteolytic bone lesions were shown to occur in the setting of extramedullary blast crisis of CML [10], granulocytic leukemia [11], or AML [12]. Bone destruction was attributed to the invasion of leukemic myeloblasts. However, the mechanism, by which ML cells produce destructive bone lesions, and the effect of therapeutic intervention on osteolytic lesions during ML treatment are not fully understood. Therefore, an* in vivo* model of osteolytic changes induced by leukemic cells may benefit further study.

In a previous study, transformation of retrovirus driver leukemia cells with constitutively active (CA) STAT5 signaling was employed to achieve second hit and leukemic transformation ability [13]. The aim of this study was the development of an* in vivo* model of osteolytic lesions induced by leukemic cells. To that end, HL-60 cells were transformed by retrovirus-mediated CA-STAT5 expression and injected into nonobese diabetic (NOD)/SCID mice via the tail vein before histocytochemical analysis of lumbar spines after a period of three weeks.

## 2. Materials and Methods

### 2.1. Cells Culture

HL-60 cells were provided by the Sun Yat-sen Institute of Hematology (Guangzhou, China). The cells were cultured in RPMI 1640 medium (Gibco, Thermo Fisher Scientific Inc., Waltham, MA) supplemented with 10% (v/v) fetal bovine serum (FBS; HyClone, Logan, UT).

### 2.2. Retroviral Infection

Retroviruses were produced from the packaging cell line GP2-293 by means of a pseudo-envelope vector. The retroviral vectors for virus packaging (MIG and MIG-STAT5CA) were gifts of Professor Richard Moriggl (University of Veterinary Medicine, Vienna, Austria) [14]. The retroviral supernatant was collected on days two and three following transfection. HL-60 cells were plated onto RetroNectin®-coated 24-well plates (Takara Shuzo, Shiga, Japan) and exposed to the retroviral supernatant in the presence of polybrene. Successful transfection was confirmed by mRNA and protein expression analysis.

### 2.3. Cell Viability Assay

Cell viability was evaluated in triplicate using the trypan blue exclusion method.

### 2.4. Colony Formation Assay

The cells were plated at 50 cells/ml in methylcellulose (R&D) using a cytokine-independent method [15]. On day 12 after plating, the colony-forming units (CFUs) were counted in three independent experiments performed in triplicate.

### 2.5. Western Blot Analysis

The cells were lysed in RIPA buffer. The protein concentration was determined by the Bradford method with BSA (Sigma) as the standard. Equal amounts of cell extract were subjected to electrophoresis in SDS-polyacrylamide gel and then transferred to a nitrocellulose membrane (Merck Millipore). The membrane was blocked and then incubated with GAPDH (Ambion, Thermo Fisher Scientific Inc.), phospho-STAT5 (Tyr694) (pY-STAT5) (Cell Signaling Technology, Danvers, MA), STAT5A, and c-Myc antibodies (Santa Cruz Biotechnology, Dallas, TX) at 4°C overnight. This was followed by incubation for 1 hr with appropriate secondary antibodies. Antibody binding was detected with an enhanced chemiluminescence kit (Pierce, Thermo Fisher Scientific Inc., Waltham, MA).

### 2.6. Real-Time PCR

Total RNA was extracted using TRIzol reagent (Invitrogen, Thermo Fisher Scientific Inc.). After reverse transcription of the total RNA, the first-strand cDNA was used as a template for detecting* STAT5A* and* MYC* expression with real-time PCR (RT-PCR) reagent from Toyobo Co. (Osaka, Japan). The primers were 5′-TGCCATTGACTTGGACAA-3′ and 5′-GTCTGGTTGATCTGAAGGT-3′ for* STAT5*, 5′-AGGAACAAGAAGATGAGGAAGA-3′ and 5′-CTGCGTAGTTGTGCTGATG-3′ for* c-Myc*, and 5′-GCGTCGTGATTAGTGATGATGA-3′ and 5′-GCACACAGAGGGCTACAATG-3′ for* HPRT1*. The latter was used as an internal control.

### 2.7. Tumor Xenograft Experiments

Male nu/nu BALB/c mice were bred at the animal facility of the Third Affiliated Hospital of Sun Yat-sen University (Guangzhou, China). The leukemia cells were introduced via subcutaneous inoculation into the flanks of 5- to 6-week-old mice. Two weeks after inoculation, the mice were euthanized, and xenografts were dissected. All animal studies were approved by the Third Affiliated Hospital of Sun Yat-sen University Institutional Animal Care and Use Committee.

### 2.8. *In Vivo* Leukemia Model with Spinal Bone Destruction

All procedures involving the use and care of animals were performed in accordance with animal standard procedures and were approved by the Institutional Animal Care Use. All experiments involved unconditioned 6- to 8-week-old NOD/SCID mice (Beijing Vital River Laboratory, China) housed under positive pressure in individually ventilated cages. The* in vivo* model was established after injection of 5 × 10^5^ transformed HL60 cells by the tail vein method. Before injection, the NOD/SCID mice were irradiated with a dose of 1.0 Gy using an X-ray generator. After 1 week, peripheral white blood cells (PWBCs) were analyzed using flow cytometry to detect surviving human leukemia cells. The animals developed hind limb paralysis in 2-3 weeks. These mice were euthanized at 3 weeks, and the lumbar spines were harvested for analysis.

### 2.9. Tartrate-Resistant Acid Phosphatase (TRAP) Cytochemistry

The lumbar spines were harvested from NOD/SCID mice, fixed in formalin, decalcified with EDTA, and embedded in paraffin. Five-micrometer deparaffinized sections were immersed in acetone and stained for TRAP according to the manufacturer's instructions (Sigma). The osteoclast measurement was performed on multiple TRAP stained sections taken at least 25 microns apart. The data was pooled to generate the derived data.

### 2.10. Statistical Analysis

Statistical analysis was performed using SPSS version 16.0 (SPSS Inc.). Student's *t*-test and chi-square test was used for statistical comparison between two groups. The level of significance was set at *p* < 0.05.

## 3. Results and Discussion

### 3.1. Constitutively Active STAT5 Signaling Promotes Malignant Transformation in Myeloid Leukemia Cells, Showing Enhanced Colony-Forming Ability and Increased Proliferative Capacity* In Vitro* and* In Vivo*

**Figure 1:**
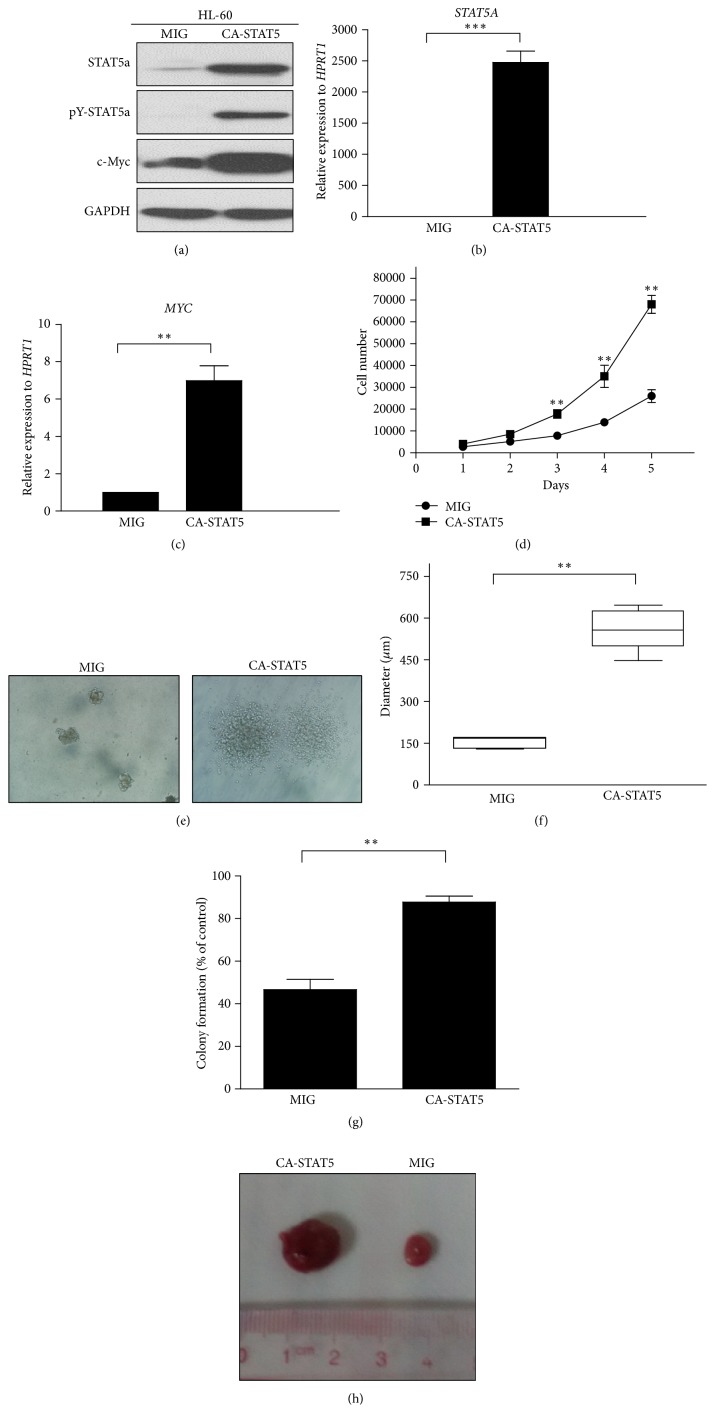
*Constitutively active STAT5 signaling promotes malignant transformation in myeloid leukemia cells, showing enhanced colony-forming ability and increased proliferative capacity in vitro and in vivo. Stable HL-60 leukemia cells with enforced expression of CA-STAT5 were generated by retrovirus infection*. (a) Western blot analysis of the expression of STAT5A, pY-STAT5a, and c-Myc. The cell extracts were probed with antibodies against STAT5A, pY-STAT5a, c-Myc, and GAPDH (loading control) as indicated. (b, c) The qPCR analyses of the expression of* STAT5A* and* c-Myc* in the stable HL-60 cells. (d) Proliferation of the stable HL-60 cells evaluated by the trypan blue exclusion method. HL-60/MIG-GFP and HL-60/MIG-STAT5CA-GFP cells were analyzed using a colony formation assay. Morphological image (e), colony size (f), and rate of colony formation (g) are shown. The HL-60/GFP and HL-60/CA-STAT5 cells were resuspended and injected subcutaneously into both sides of the backs of male nude mice (BALB/C, nu/nu) to establish a human leukemia xenograft model. (h) Representative tumors removed from mice are shown. The results are shown as the mean values ± SD of 3 independent experiments. ^*∗*^*p* < 0.05, ^*∗∗*^*p* < 0.01, compared to the control.

Using retrovirus infection and flow sorting of GFP-expressing cells, stable HL-60 leukemia cells with enforced expression of a constitutively active form of the* STAT5A* gene (CA-STAT5) were generated. Aberrant expression of CA-STAT5 in HL-60 cells upregulated the STAT5 signaling-related downstream target, c-Myc, at both the transcriptional and the protein level (Figures 1(a)–1(c)), thus contributing to leukemogenesis by preventing apoptosis and promoting cell proliferation [16, 17] as evidenced by a time-dependent increase in cell count (Figure 1(d)). In addition, CA-STAT5 enhanced colony formation, generating larger spheres (Figures 1(e) and 1(f)). The rates of colony formation were 48.65% ± 1.28% and 89.83% ± 0.73% in control and CA-STAT5 cells, respectively (Figure 1(g)). Similar results were shown in other myeloid leukemia cells, such as NB4 and U937 cells (data not shown). For* in vivo* tumor xenograft experiments, the recipient mice transplanted with CA-STAT5 leukemia cells developed larger tumors than control mice (*n* = 3) (Figure 1(h)). Our results suggest that high cellular STAT5 activity promotes uncontrolled proliferation of leukemia cells.

STAT5 can be activated by various cytokines, growth factors, and genetic factors. STAT5 signaling is required for the efficient induction and maintenance of CML [18]. BCR/ABL fusion protein, a feature of CML cells, causes the activation of STAT5 and leads to increased expression of genes that drive cell cycle progression and promote survival [19]. STAT5 signaling is also aberrantly active in some forms of AML. Numerous studies have supported the notion that STAT5 signaling acts as a diagnostic or prognostic marker for myeloid leukemia [20]. However, it has not yet been fully determined how a second hit directly augments STAT5 activity in ML cells or drives the progression of disease symptoms. Collectively, our results show that direct activation of STAT5 signaling may increase the malignancy of myeloid leukemia cells, which show enhanced colony formation and increased proliferative capacity* in vitro *and* in vivo*. Our data suggest that the direct activation of STAT5 signaling in ML cells may produce malignant disease symptoms during leukemogenesis, including extramedullary invasion.

### 3.2. Constitutively Active STAT5 Signaling Induces Spinal Bone Destruction by Extramedullary Invasion of Myeloid Leukemia Cells* In Vivo*

**Figure 2:**
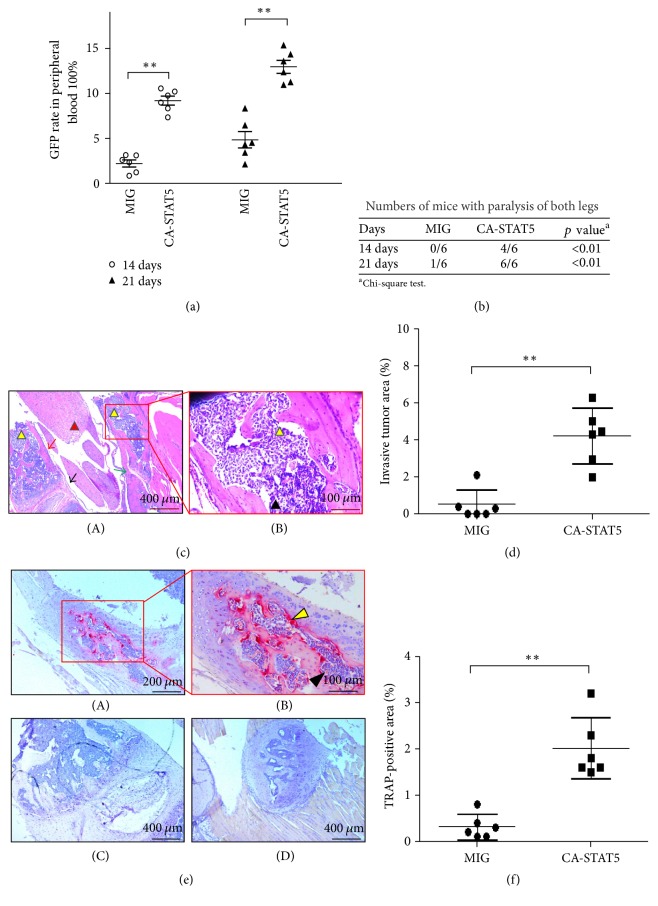
*Constitutively active STAT5 signaling induces spine bone destruction by extramedullary invasion of myeloid leukemia cells in vivo*. The HL-60 leukemia cells with control or CA-STAT5 overexpression were injected into the NOD/SCID recipient mice irradiated with 1 Gy using the tail vein method. (a) Two or three weeks after transplantation, the PWBCs were counted by flow cytometry. (b) Statistical analysis of mice with paralysis of both legs. *p* value was computed by the chi-square test. (c) Representative images of H&E stained sections of the lumbar spines from the CA-STAT5 mice from low (A) to high (B) magnification. In (c) (A), the red arrow indicates the vertebral body, the black arrow the intervertebral disc, the green arrow the lamina joint, the red triangular region the spinal cord, and the yellow triangular region the leukemic cells. In (c) (B), leukemic cells, unlike normal bone marrow cells, showed irregular shapes, large nuclei, and shallow staining. The yellow arrow indicates the leukemic cells, and the black arrow indicates normal bone marrow cells. (d) Relative invasive tumor area was measured by spinal histomorphometry using H&E stained sections taken at least 25 microns apart. (E) TRAP cytochemical analysis of the representative sections of the lumbar spines from the CA-STAT5 mice (A, B) and control (C, D) from low to high magnification. Invasion of leukemic cells into the spinal bone environment enhanced significantly osteoclast activity (*p* < 0.01). Deeper red staining represents higher activity. TRAP staining showed no osteoclast activation in the control group. The yellow arrow indicates focal accumulation of leukemic cells, and the black arrow indicates normal bone marrow cells. (f) The osteoclast measurement was performed on multiple TRAP stained sections taken at least 25 microns apart. The data was pooled to generate the derived data. ^*∗*^*p* < 0.05, ^*∗∗*^*p* < 0.01, compared to the control.

Stable HL-60 leukemia cells with control or CA-STAT5 overexpression were injected into the irradiated NOD/SCID recipient mice via the tail vein. After 2 or 3 weeks the peripheral white blood cells (PWBC) were analyzed by flow cytometry.  Figure 2(a) shows that high STAT5 activity increases the survival and proliferation of leukemia cells* in vivo*. After 3 weeks, the percentage of the exogenous leukemia cells identified as GFP-positive cells was 12.95% ± 0.72% in the CA-STAT5 group (*p* < 0.001; GFP group: 4.84% ± 0.91%), indicating that all mice in the CA-STAT5 group developed xenograft myeloid leukemia (*n* = 6). Moreover, every mouse in the CA-STAT5 group (6/6) developed hind limb paralysis 3 weeks after injection (Figure 2(b)). In contrast, only a single mouse in the control group (1/6) experienced hind limb paralysis (Figure 2(b)). These results suggested that CA-STAT5 signaling was capable of spinal bone destruction* in vivo*. To verify bone destruction, the lumbar spines of the CA-STAT5 mice were harvested for H&E staining and TRAP cytochemistry. Histological analysis showed a significant increase in the number of leukemia cells in the spinal lamina (*p* < 0.001), suggesting that the CA-STAT5 ML cells had migrated to and invaded the lamina (Figures 2(c) and 2(d)). TRAP activity may reflect osteoclast activation [21]. As demonstrated by TRAP staining, osteoclast activity in the bone environment was obviously enhanced in the CA-STAT5 group (Figure 2(e)). TRAP activity was not detected in the normal control group (Figure 2(e)). Statistical analysis also revealed an increased number of active osteoclasts in the spinal lamina (*p* < 0.0001) (Figure 2(f)), suggesting that osteoclast activation is due to the invasion of ML cells into the cartilage. In other words, the CA-STAT5 ML cells may act on the spinal bone environment to induce osteolytic lesions and bone destruction.

Bone destruction is attributed to the invasion of leukemic myeloblasts or an imbalance of osteoblasts and osteoclasts. An increased number and activity of osteoclasts contribute to bone marrow necrosis or osteolytic lesions [22]. STAT5-induced cell proliferation per se is not the cause of osteocyte destruction. Rather leukemia cells with high STAT5 signaling may secrete specific factors to activate osteoclasts leading to the breakdown of bone tissue. Elucidation of the exact molecular mechanism will be subject of further study.
